# A review of SaiLuoTong (MLC-SLT) development in vascular cognitive impairment and dementia

**DOI:** 10.3389/fphar.2024.1343820

**Published:** 2024-05-01

**Authors:** Encarnita Raya Ampil, Paulus Anam Ong, Yakup Krespi, Yuan-Han Yang

**Affiliations:** ^1^ Department of Neuroscience and Behavioral Medicine, Faculty of Medicine and Surgery, University of Santo Tomas, Santo Tomas, Philippines; ^2^ Institute for Neurosciences, St. Luke’s Medical Center-Global City Philippines, Taguig, Philippines; ^3^ Department of Neurology, Hasan Sadikin Hospital, Universitas Padjadjaran, Bandung, Indonesia; ^4^ Department of Neurology, İstinye University Hospital, Istanbul, Türkiye; ^5^ Department of Neurology, Kaohsiung Municipal Ta-Tung Hospital, Kaohsiung Medical University Hospital, Kaohsiung, Taiwan

**Keywords:** cognivaid, MLC-SLT, sailuotong, vascular cognitive impairment, vascular cognitive impairment and dementia, vascular dementia, VaD, VCID

## Abstract

The dementia epidemic, attributed to aging populations, represents a growing socio-economic burden. It is estimated that in 2019 about 55 million people worldwide were living with dementia. With many possible causes of dementia and the possibility of mixed dementia combining Alzheimer’s disease (AD) and vascular dementia the question is whether diagnostic uncertainty exists or whether diagnostic constructs based on single etiologies are incorrect. Vascular Cognitive Impairment and Dementia (VCID) designates the extent of cognitive dysfunctions from the most benign state to that of dementia, of vascular origin. We reviewed epidemiological, pathophysiological and clinical data on VCID with a focus on VaD, as well as key data on the development of a new therapeutic solution, SaiLuoTong (MLC-SLT). From documentary research executed on different web sources (PubMed, Clintrials.gov, Z-library and Google), our initial selection for the short review of VCID and VaD was based on keywords contained in each paragraph subtitles of this article with exclusion of publications in a language other than English or published before 2010. For the review of SaiLuoTong development, there was just the language exclusion criterion. Sorted by relevance and publication date, 47 references were selected from 140 shortlisted for review. With new evidence-based classification systems, vascular cognitive impairment was proposed as umbrella term covering all forms of cognitive deficits related to vascular causes. The scope of application expanded with the VCID which includes VaD and mixed pathologies. No drugs are approved for the treatment of VaD by major Western regulatory agencies, while some traditional Chinese medicines are registered in China. VCID treatment should have a dual focus: managing the underlying cerebrovascular disease and dementia symptoms. This is the objective set for the development of the MLC-SLT, the essential data of which are reviewed in detail. To strengthen VCID and VaD research, consensus groups should attempt to consolidate scattered local research initiatives into coordinated international programs. In two VaD clinical trials, MLC-SLT improved cognitive symptoms and activities of daily living, with good safety and potential disease-modifying effect. In a placebo-controlled study in 325 patients with mild to moderate VaD and randomized according to a delayed-start design, MLC-SLT demonstrated significant improvement in memory tests and performance in executive function tasks, expanding its place in the management of VCID. At week 26, changes in VADAS-cog scores (SD) from baseline were 23.25 (0.45) for MLC-SLT 180 mg bid), 23.05 (0.45) for MLC-SLT 120 mg bid (both *p* < 0.0001), and 20.57 (0.45) for placebo (*p* = 0.15). At week 52, differences between both groups MLC-SLT and placebo were 2.67 and 2.48, respectively (*p* < 0.0001), without significant difference between MLC-SLT groups.

## 1 Introduction

Dementia is a syndrome of progressive chronic cognitive decline leading to functional impairment (Am Psychiatric Association, 2013). In the Diagnostic Manual of Mental Disorders, Fifth Edition (DSM-5), cognitive decline is quantified as deficits occurring in one or more domains (e.g., memory, executive function, visuospatial, language, attention) (Sachdev et al., 2014). It is estimated that in 2019 about 55 million people worldwide were living with dementia (Western Pacific Region: 20.1 million, European Region: 14.1 million, Region of the Americas: 10.3 million, South-East Asia Region: 6.5 million, Rest of the World: 3.4 million) (World Health Organization, 2021). Assuming no change in the age-specific prevalence rates over the next decades, World Health Organisation projects around 78 million people with dementia worldwide in 2030 and about 139 million in 2050. This upcoming dementia epidemic is attributable to rapidly ageing populations and will present an increasing socio-economic burden. According to new evidence-based classification systems, the term vascular cognitive impairment (VCI) has been proposed by various authors as an umbrella term covering all forms of cognitive deficits that are clinically attributed to a vascular cause, from vascular mild cognitive impairment (MCI) up to vascular dementia (VaD) (O'Brien et al., 2003; Jellinger, 2013; Calabrese et al., 2021). Expert groups have agreed to rename this diagnostic continuum as vascular contributions to cognitive impairment and dementia (VCID), comprising a broad range of vascular mechanisms and phenotypes found within this heterogeneous group of disorders (Zlokovic et al., 2020). Diagnosis of the VCID spectrum should be based on neuropsychological testing, clinical examination, and neuroimaging.

Thus, we aimed to review the properties of a new herbal formulation, named SaiLuoTong (MLC-SLT), having demonstrated promising pharmacological effects on both cerebrovascular and neurodegenerative components of VCID, which support its clinical development in the management of subjects suffering from VCID, particularly those with mild to moderate VaD.

## 2 Method

According to the PRISMA checklist 2020 (http://prisma-statement.org/PRISMAStatement/Checklist), we used the following selection criteria for our literature search about VCID, VaD, VCI, and MCI:

• Inclusion criteria: animal studies; clinical studies in subjects or patients with VCID or VaD or VCI or MCI; placebo-controlled experimental preclinical and clinical studies; observational and epidemiological studies; recent publications since 2010. However, we have included certain older publications that we considered to report key steps in the management of the pathologies concerned by this review.
• Exclusion criteria: publications in a language other than English.

For the search about MLC-SLT and its ingredients, we included all the publication about preclinical or clinical studies containing sailuotong or weinaokang AND VCID or VaD or VCI or MCI. For the review of SaiLuoTong development there was only a language other than English exclusion criterion.

We searched PubMed, Clintrials.gov, Z-library and Google for reviews and original articles published in English using the following keywords (singular or in a phrase): “cognitive impairment,” “vascular cognitive impairment,” “dementia,” “vascular dementia,” “Alzheimer’s,” “new drugs,” “pharmacology,” “clinical development,” “sailuotong,”“TCM,” “weinaokang.” We also furthered our search by scanning the bibliographies of systematic review articles. For this review, we have selected the most relevant and most recent articles for vascular cognitive impairment and dementia (VCID), using the keywords contained in the subtitles of each corresponding paragraphs of this article. Therefore, after screening 140 articles, 47 references were selected for this review.

## 3 Vascular cognitive impairment and dementia

VCI refers to a composite group of conditions whereby vascular factors may be associated with or be a cause of vascular brain injury, leading to cognitive deficits. While atherosclerotic and cardioembolic diseases are common causes of vascular brain injury, cerebral small vessel disease (CSVD) emerges as a core substrate of VCI (Kalaria, 2016). CSVD is characterized by arteriolosclerosis, lacunar infarcts, and cortical and subcortical microinfarcts, with diffuse white matter lesions (WMLs) leading to loss of myelin and axonal abnormalities. In addition to single strokes, multiple infarctions and vascular white matter injury, the term VCI also considers other cerebrovascular disease mechanisms such as chronic cerebral hypoperfusion responsible for a continuum of cognitive deficits associated with vascular cognitive impairment and dementia (VCID) (Frantellizzi et al., 2020).

The diagnosis of VCID is based on a thorough history and physical examination, focusing on cognitive and functional deficits, their onset and symptomatic progression, and on the search for history of vascular brain injury such as stroke or CSVD, and of vascular risk factors such as hypertension, diabetes, hyperlipidaemia, smoking, obesity, and sedentary lifestyle (Simmons et al., 2011). With magnetic resonance imaging (MRI) allowing identification of vascular injury, the diagnosis of VCID can be made with certainty. However, given the many possible causes of dementia, and the possibility of a mixed dementia syndrome combining AD with VaD, the question is whether there is diagnostic uncertainty or whether diagnostic constructs based on single etiologies are incorrect (Gorelick et al., 2011). So, it may not be sufficient in itself to fully elucidate a more comprehensive and multi-etiology diagnosis. Epidemiological evidence supports a convergence of pathogenic mechanisms in vascular and neurodegenerative processes of VCID that cause impairment of cognition.

VaD is widely recognised as the second most common type of dementia (15%–20% of dementia cases in North America and Europe and around 30%, in Asia and developing countries) (O'Brien and Thomas, 2015; Wolters and Ikram, 2019), just after Alzheimers’ disease (60%–80% of all dementia cases) (Gustavsson et al., 2023). However, subjects with mixed degenerative and vascular pathology have nearly twice the incremental risk of dementia (77%–86%) compared with those having only pure degenerative pathology of Alzheimer-type (∼45%) (Azarpazhooh et al., 2018).

## 4 Management of VCID

A recent scientific expert panel from the Journal of the American College of Cardiology (JACC) provides a critical appraisal of the epidemiology, pathobiology, neuropathology, and neuroimaging of VCID, and of current diagnostic and therapeutic approaches (Iadecola et al., 2019).

There are currently no drugs approved specifically for the treatment of VaD by the main Western regulatory agencies (US FDA and EMA), while Traditional Chinese Medicine (TCM) is registered in China with this indication. However, preclinical studies have explored a range of potential therapeutic targets for VCID including inflammation, oxidative stress, and neurovascular coupling (Sun, 2018). The therapeutic approach in VCID patients should have multiple objectives managing the underlying cerebrovascular disease, as well as the associated cardiovascular risk factors (CVRFs), and addressing dementia symptoms (Yu et al., 2022).

Concerning the management of CVRFs, disease-related dementia risk might be better captured by considering all the CVRFs together using the Framingham Risk Score rather than individually (hypertension, diabetes, obesity, smoking, *etc.*) (Farnsworth von Cederwald et al., 2022). Hence, all these factors should be treated with medications and appropriate lifestyle changes, as recommended in the Guideline on covert cerebral small vessel disease of European Stroke Organisation (ESO) (Wardlaw et al., 2021) and the recommendations from the JACC Scientific Expert Panel (Iadecola et al., 2019).

Regarding the cognitive impact of VCID, clinical trials have been conducted with drugs targeting the cholinergic system, such as choline esterase inhibitors (ChEIs) and memantine, an N-methyl-D-aspartate receptor antagonist. A recent Cochrane meta-analysis concluded with moderate to high-certainty evidence that donepezil 5 mg and 10 mg, and galantamine have a limited beneficial effect on cognition in people with VCI, the magnitude of which is probably not clinically significant (Battle et al., 2021). Additionally, donepezil 10 mg and galantamine 16–24 mg are associated with more adverse effects than placebo. Overall, the evidence for specific treatments for VaD is limited, with further research being needed to identify effective therapies.

Since the accumulation of amyloid β (Aβ) peptide has been one of the most studied pathophysiological mechanisms in the field of dementia for many years, several other molecular mechanisms involved in pathogenesis of VCID could be the target of new treatments (Kuang et al., 2021). This is the case for synaptic damage and/or synaptic loss, functional restoration of blood-brain barrier disruption, stimulation of expression and activity of brain derived neurotrophic factor (BDNF), enhancing neurogenesis, activation of an antiapoptotic effect, phosphorylation of mitochondrial K+ATP channel, and underdiagnosed WMLs. The close interactions between these mechanisms further contribute to the difficulty of diagnosing and treating VCID.

**FIGURE 1:**
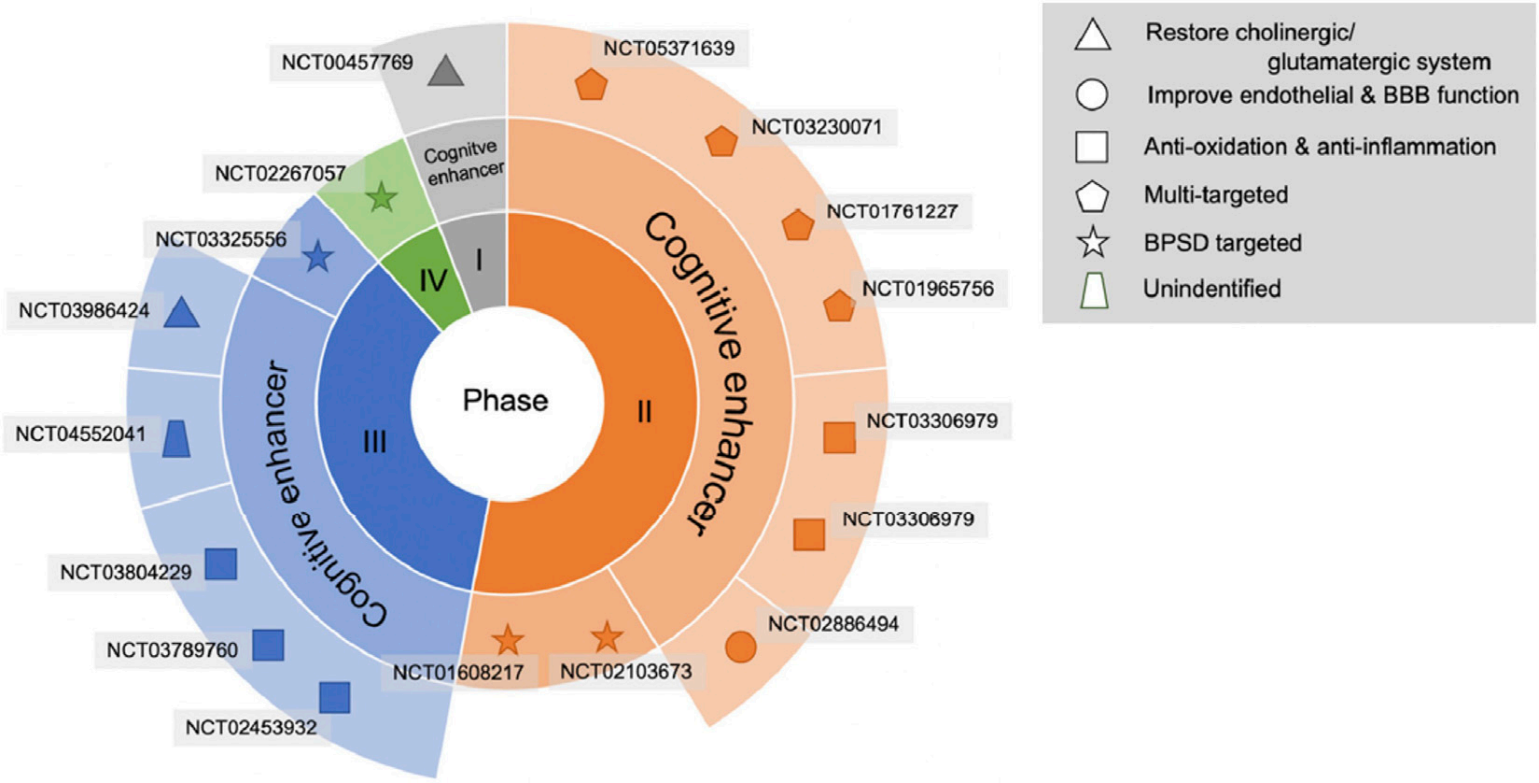
Drug development pipeline in VCI or VaD treatment. The agents are displayed using specific shapes corresponding to their pathophysiological mechanisms. Abbreviations: BBB, blood–brain barrier; BPSD, behavioural and psychological symptoms of dementia.^©^ 2022 by the authors. Licensee MDPI, Basel, Switzerland. This article is an open access article distributed under the terms and conditions of the Creative Commons Attribution (CC BY) license (https://creativecommons.org/licenses/by/4.0/).

In clinical trials, VaD, the ultimate stage of VCID, is the stage most often used to test new treatments. A recent literature search on ClinicalTrials.gov identified five phase 3 trials investigating cognitive function enhancers, of which 3 are ongoing, 1 completed without published results, and 1 published (Linh et al., 2022) (Figure 1). The published study was conducted in China (NCT02453932) with a traditional herbal drug, named Tianzhi (TZ) granules (5 g, 3 times per day), compared to donepezil (5 mg per day) and placebo, during 24 weeks (Shi et al., 2020). The results showed that both TZ and donepezil have similar small treatment effects at 24 months, which were significantly higher compared to the placebo group, as measured on Vascular Dementia Assessment Scale-cognitive (VADAS-Cog) and Clinician’s Interview-Based Impression of Change (CIBIC)-Plus scores. A limitation of this study is that the placebo group (*n* = 60) was about 4-fold smaller than the TZ and donepezil groups (*n* = 242 and 241, respectively). A new multi-herbal formulation, SaiLuoTong, trade name CognivAiD™, and international non-proprietary name (INN) MLC-SLT, was subject to a preclinical and clinical development program having allowed its marketing authorisation and the review of which constitutes the main objective of this article.

## 5 SaiLuoTong (MLC-SLT) development outcomes

Since April 2022, SaiLuoTong (MLC-SLT) has been listed in Singapore as a Chinese Proprietary Medicine by the Health Sciences Authority (HSA) with the brand name CognivAiD™ and recommended to help improve age-related mental and cognitive abilities such as memory, behaviour and concentration. Clinically, key findings include studies of MLC-SLT in VaD and preliminary data in MCI, the whole matching the continuum of cognitive deficits associated with VCID.

### 5.1 MLC-SLT composition

MLC-SLT is a novel standardized formula combining specific doses of three bioactive compounds derived from concentrated extracts of Panax ginseng, Ginkgo biloba and Crocus sativus. Given the known pharmacological properties of these compounds, it has been developed to improve cognitive and cerebrovascular functions through multiple pathways of action.

#### 5.1.1 Panax ginseng

Used in China for thousands of years in the treatment of dementia, Panax ginseng (Pg) contains many active compounds, such as ginsenosides, polysaccharides, amino acids, volatile oils and polyacetylenes, many of which have beneficial effects in the treatment of dementia. For example, ginsenosides act on various targets involved in the pathophysiology of dementia, such as the regulation of synaptic neuroplasticity and the cholinergic system, the inhibition of Aβ peptide aggregation and of hyperphosphorylation of tau, anti-inflammatory properties, as well as antioxidant and anti-apoptosis effects. The other active components of Pg, such as gintonin, oligosaccharides, polysaccharides and ginseng proteins, also have documented promising effects on AD and VaD (Wang et al., 2023). The favourable effects of TCM containing ginseng have also been reported in preclinical and clinical studies for the treatment of AD and VaD.

#### 5.1.2 Ginkgo biloba

Well known all over the world, Ginkgo biloba (Gb) contains flavonoids, terpene lactones and various other biological components (Biernacka et al., 2023). Gb neuroprotective properties and favourable pharmacological effects on cognitive and neurological functions are attributed to the improvement of blood flow by inducing nitric oxide production, inhibition of platelet activating factors, its antioxidant effect, anti-inflammatory and anti-apoptotic actions, improvement of neuroplasticity, modulation of Aβ peptide aggregation and protection against mitochondrial dysfunction. In clinical trials, some favourable effects of Gb have been shown in VaD patients (García-Alberca et al., 2022; Cui et al., 2023). In a small retrospective study with 77 VaD patients divided into 3 groups, Gb (EGb761) combined with an AChEI is better than each individual treatment by increasing significantly cognitive and behavioural benefits after 12 months of treatment (García-Alberca et al., 2022). A systematic review of Gb in MCI and dementia concluded with strong evidence to support the efficacy of Gb extracts in MCI and dementia; however prophylactic effects on cognitive decline are less conclusive (Hort et al., 2023).

#### 5.1.3 Crocus sativus (Saffron)

Crocus sativus (Cs), which is Saffron, has been used as a spice, dye and herbal medicine for a long time. Its beneficial effects on the cognitive performance were shown in patients with MCI and AD as reported in a systematic review and meta-analysis of randomized clinical trials (RCTs), but no studies about its effects in VaD were found (Ayati et al., 2020). Its major bioactive compounds are crocin, crocetin, safranal and picrocrocin (D'Onofrio et al., 2021). The exact mechanisms underlying the effects of Cs on dementia remain unclear, but preclinical studies support its anti-atherosclerotic properties, as well as its antioxidant, anti-inflammatory, anti-convulsive, anti-nociceptive, anti-depressant, and anxiolytic effects (El Midaoui et al., 2022).

### 5.2 Preclinical development

In the preclinical studies on MLC-SLT, the main objective was to demonstrate its multi-target mechanism of action due to its multiple herbal components working synergistically to improve cerebral perfusion and alleviate cognitive impairment. We will review the pharmacological studies which validate the effects of MLC-SLT on vasculature protection, vascular reactivity enhancement, and neuroinflammation modulation.

#### 5.2.1 Vasculature protection

The cerebral endothelium and microvessels have been suggested to support neuronal functions and homeostasis. A study explored the cellular mechanisms underlying the protective effect of MLC-SLT on the cerebrovascular system (Seto et al., 2017).

MLC-SLT has been found to protect the cerebral endothelium and microvessels, which support neuronal functions and homeostasis, and it significantly reduces reactive oxygen species (ROS) levels and reverses the reduction in superoxide dismutase (SOD) activity. It also inhibits apoptosis of human endothelial cells and reduces the increase in cleaved caspase-3 expression. The effect of MLC-SLT on oxidative stress resulting from acute ischemia was assessed using a hydrogen peroxide (H2O2) model in human endothelial cells. The presence of H2O2 induced a 2-fold increase in ROS. ROS levels were significantly reduced in the MLC-SLT groups (*p* < 0.001 vs. Sham) in a concentration-dependent manner.

#### 5.2.2 Vascular reactivity enhancement

Altered vascular reactivity, such as impaired vasodilation or enhanced vasoconstriction, plays a role in the development and progression of cerebrovascular disease. This study aimed to examine the vasodilatory effects of MLC-SLT and the underlying mechanisms in rat isolated tail artery (Yeon et al., 2020). MLC-SLT induced vasodilatation via an endothelium-independent mechanism, meditated by jointly blocking the extracellular Ca2+ influx and the release of intracellular Ca2+ stores.

This study showed that the vasodilatory effect of MLC-SLT was mediated by an endothelium-independent mechanism jointly blocking the extracellular Ca2+ influx and the release of intracellular Ca2+ stores. The vasodilatory effect was sustained and concentration-dependent, irrespective of the presence of an endothelium derived vasodilator (nitric oxide) inhibitor (l-N-nitro arginine methyl ester–L-NAME). The vasodilatory effect was not mediated by potassium channels, and the study also revealed that MLC-SLT reduced the release of stored intracellular Ca2+ by inhibiting the release of stored intracellular Ca2+.

#### 5.2.3 Neuroinflammation modulation

Brain injury or infection can result in neuroinflammation that may encourage the release of lipocalin 2 (LCN2) from astrocytes. High levels of LCN2 aggravate neuroinflammation and may later reduce cognitive activity, making LCN2 a possible target in anti-inflammation therapies. This study aimed to investigate how MLC-SLT regulates astrocyte activities through its anti-inflammatory effects and in so, reduces LCN2 overexpression. Here we will summarize the main reported results (Zhang et al., 2019).

**FIGURE 2:**
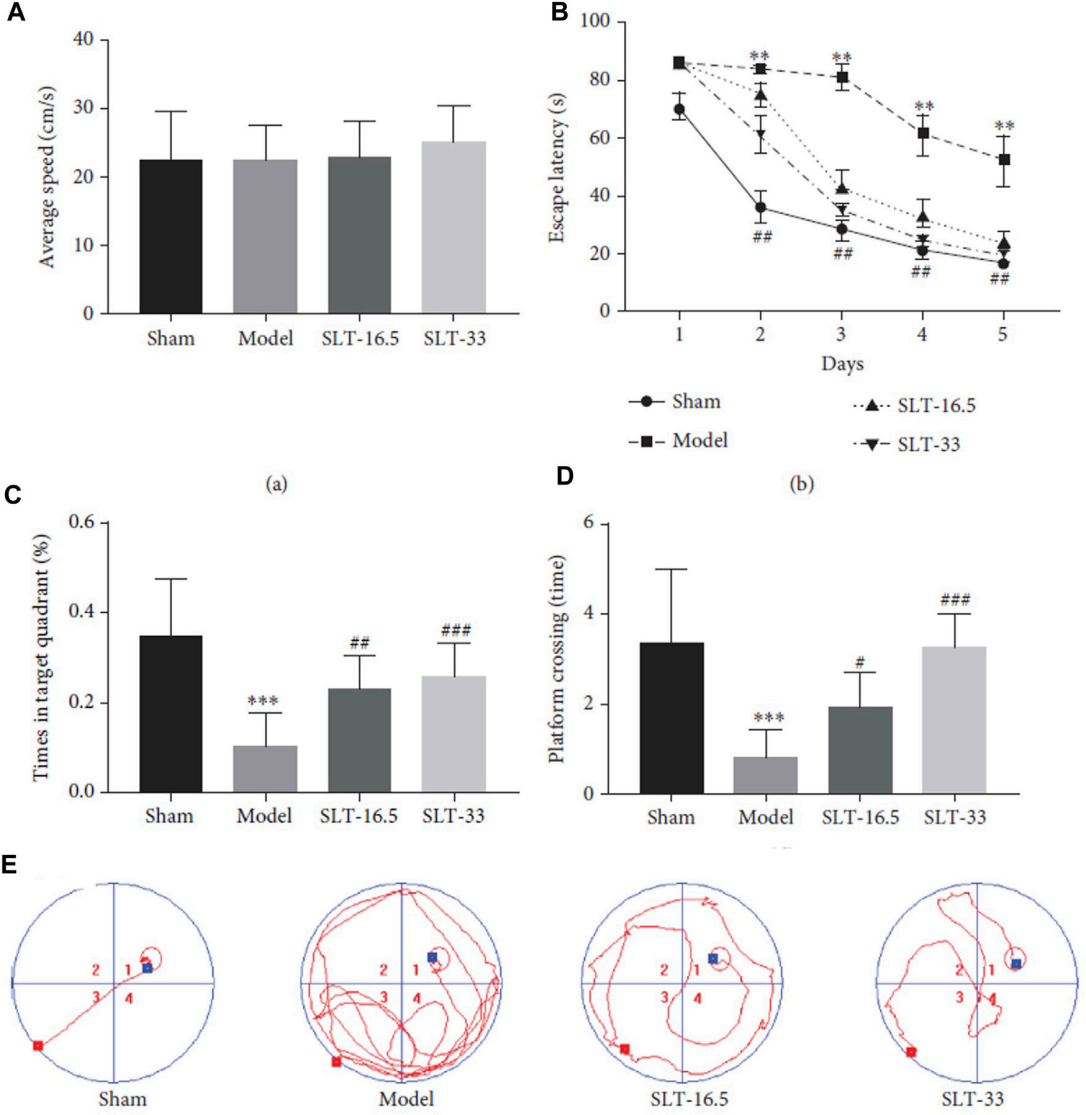
Neuroprotective effects of SLT on the Morris water maze (MWM) test. **(A)** average swimming speed, **(B)** escape latency, **(C)** platform crossing, **(D)** target quadrant time, and **(E)** trajectory of swimming. Data are expressed as the mean ± SEM (*n* = 10). ∗∗*p* < 0 01 and ∗∗∗*p* < 0 001 vs. sham group; #*p* < 0 05, ##*p* < 0 01, and ###*p* < 0 001 vs. model groups. ^©^ 2022 by the authors. Licensee MDPI, Basel, Switzerland. This article is an open access article distributed under the terms and conditions of the Creative Commons Attribution (CC BY) license (https://creativecommons.org/licenses/by/4.0/).

This study demonstrated that MLC-SLT can modulate neuroinflammation, reducing the risk of brain injury, by regulating astrocyte activities and reducing LCN2 overexpression in the ischaemic hemisphere. The administration MLC-SLT for 28 days, resulted in lower neurological deficit scores and brain tissue lesions, and improved memory retention and reduced inflammation markers. In an oxygen-glucose deprivation (OGD) ischaemia model, MLC-SLT negated the overexpression of LCN2 after OGD induction. The effect of MLC-SLT on memory impairment induced by cerebral ischaemia was measured with the Morris Water Maze test, showing that for both MLC-SLT groups, the time spent in the target quadrant was greater than that of the model group (*p* < 0.001), reaching even values observed in the sham group (Figure 2). These results suggest that MLC-SLT has antiastrogliosis and antineuroinflammatory effects, improving neuronal survival and memory deficiency in *in vitro* and *in vivo* models of ischaemia. As a multiherbal formulation, MLC-SLT may apply for neuroinflammatory diseases like VaD through the inactivation of LCN2 from astrocytes.

#### 5.2.4 Summary of preclinical development

**FIGURE 3:**
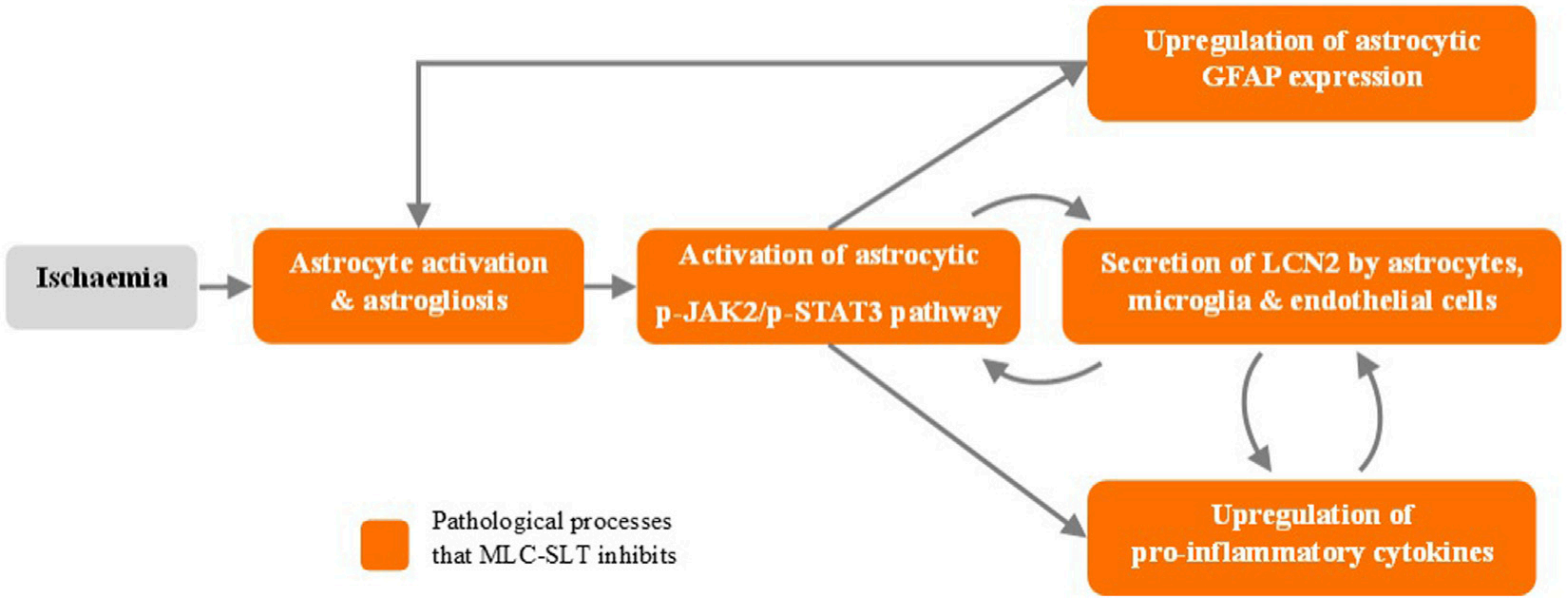
MLC-SLT effects on neuroinflammation process. p-JAK2, Phosphorylated Janus kinase 2; p-JAK2, Phosphorylated Janus kinase 2; p-STAT3, Phosphorylated Signal transducer and activator of transcription 3; GFAP, Glial fibrillary acidic protein; LCN2, Lipocalin 2.

The preclinical development outcomes of MLC-SLT in models of VCID confirm its multi-target mechanisms of action which work synergistically to improve cerebral vascularisation and alleviate cognitive impairment (Figure 3). All these favourable effects on cerebral vascular and cellular functions make MLC-SLT a promising candidate for patients with VCID, as we will see in the next chapter dedicated to the clinical development of MLC-SLT.

### 5.3 Clinical development

At first, it will be important to review the safety of MLC-SLT in human, starting with studies in healthy volunteers. Then, the randomized clinical trials will give the opportunity to estimate the benefit/risk ratio of MLC-SLT in patients with VaD, selected as the target disease in this development programme.

#### 5.3.1 Healthy volunteer studies

A study published in 2012 had the double objective to evaluate the tolerability and safety of i) single and ii) continuous dosing of SaiLuoTong capsule in healthy volunteers (Li et al., 2012). In the single dose trial, 30 subjects were randomly divided into 7 dose groups (60, 120, 180, 240, 300, 420, 540 mg). No significant changes in vital signs (heart rate, respiratory rate, blood pressure) or hepatic, renal and cardiac functions were observed in subjects who received a single dose. In the single-blind, randomized, placebo-controlled continuous dosing trial, 24 subjects were divided equally into a low dose group (180 mg t.i.d) or high dose group (300 mg t.i.d) and given intervention for 2 weeks. There was no significant difference either in the rate of symptomatic adverse events between the two groups, or in laboratory test parameters (biochemistry, haematology, urinalysis, faecal occult blood test).

To test the potential of MLC-SLT to improve cognitive and cardiovascular function, 16 healthy volunteers were recruited into a pilot randomized, double-blind, placebo-controlled crossover trial with a 1-week washout period (Steiner et al., 2016). Neurocognitive functions were assessed using a battery of computerized cognitive tests, amplitudes of event-related potential (ERP) components of the oddball task, and amplitudes of resting EEG spectral bands. Assessments of cardiovascular system functions were central and peripheral pulse pressure and heart rate at rest. It was hypothesized that 1-week administration of MLC-SLT 60 mg bid would improve neurocognitive and cardiovascular function compared to placebo. The main results are an increased speed of response in Alphabetic Working Memory Task, an improved accuracy of response in N-Back Task, and potentially, a more efficient attentional processing of auditory information and an increased activation of working memory processes. Additionally, the trial confirmed a favourable safety profile with no SAE occurrence, no difference in rate of AEs vs. placebo, and no significant difference on cardiovascular function parameters.

Overall, MLC-SLT has a favourable effect on neurocognitive function in healthy volunteers, increasing working memory performance. It is clinically safe and well tolerated, especially on cardiovascular parameters. The authors recommend to explore MLC-SLT in healthy older adults and individuals with MCI.

#### 5.3.2 Pilot trial of MLC-SLT (WeiNaoKang) in VaD

This 16-week randomized double‐blind, placebo‐controlled trial included 62 patients with a clinical diagnosis of VaD based on National Institute for Neurological Disorders and Stroke avec l'Association Internationale pour l'Enseignement et la Recherche (NINDS‐AIREN) criteria, supported by imaging (computed tomography (CT) scan or MRI, Single Photon Emission Computed Tomography (SPECT) cerebral perfusion scan) and other investigations (blood tests assessing reversible factors, and renal and liver functions). Patients receiving cholinesterase inhibitors were allowed to participate if they had no significant clinical change on Alzheimer’s Disease Assessment Scale-Cognitive subscale (ADAS‐Cog) and mini mental-state examination (MMSE) scores over the last 3 months with the medication. Study treatment capsules administered on top of cholinesterase inhibitors contained 60 mg of active ingredients, the placebo tasting smelling and looking the same as MLC-SLT ones. Primary endpoint was ADAS-Cog measuring cognitive function, the secondary endpoints being MMSE, Alzheimer’s Disease Cooperative Study–Activities of Daily Living (ADCS-ADL) for activities of daily living and Short Form Health Survey-36 (SF36) for quality of life. SPECT scans were performed at baseline and study-end in a subset of 18 randomized patients. The ratios of probable vs. possible VaD in MLC-SLT and placebo groups were 25:5 and 24:8, respectively, and that of mild vs. moderate VaD were 14:16 and 12:20, respectively (Liu et al., 2022).

The mean ADAS-Cog scores were reduced in both groups but significantly more in MLC-SLT group at the end of treatment period (−4.18 ± 0.75 vs. −1.18 ± 0.58), the mean difference between groups being 3.00 ± 0.75 (*p*-value = 0.003). As there was no clinically significant baseline difference, an analysis of covariance (ANCOVA) with the baseline ADAS-Cog as covariate was done, showing that the between-group difference at 16 weeks was still statistically significant (*p* = 0.004).

MMSE scores improved at week-16 in MLC-SLT group, but just over the statistical significance (*p* = 0.06). For the mean differences of activities of daily living (ADL) between treatment groups, despite a higher score at week-16 in MLC-SLT group (2.13 vs. 0.73 in the placebo group), the mean difference was not significant. But regarding ADCS‐ADL assessment by carers, there was a significantly higher rate of ADL improvement in MLC-SLT group vs. placebo group (63% vs. 33%, respectively; *p* = 0.027). The Short Form (SF)-36 Health Survey showed a significant improvement with MLC-SLT compared to placebo (*p* = 0.006), the overall health assessed by item 2 of SF-36 (Compared to 1 year ago, how would you rate your health in general now?) being correlated to cognitive function measured by ADAS-Cog. MLC-SLT nearly triples the proportion of subjects who reported better quality of life vs. placebo.

The difference in change in cerebral blood flow (t-scores) measured by SPECT before and after treatment in 18 patients (MLC-SLT = 7; placebo = 11), was significantly increased in the MLC-SLT group compared to placebo group (*p* < 0.001) in the inferior frontal and anterior temporal areas on both hemispheres.

The authors concluded that MLC-SLT “may be useful in treating VaD, especially in Asian countries where VaD is more prevalent than the West and in countries where cost of treatment of dementia may be an issue, this medication may also be useful as well.”

#### 5.3.3 Efficacy and safety of MLC-SLT as first-line treatment in VaD

The study protocol was published in ClinicalTrials.gov (ID: NCT01978730). This clinical trial conducted in 16 academic centres in China included 325 patients with probable mild to moderate VaD diagnosis treated according to a delayed-start design and with two daily dose schedules of MLC-SLT (240 mg or 360 mg divided into 2 doses per day) after a 4-week placebo run-in period (Jia et al., 2018). Patients were randomly assigned in double-blind between four arms, of which two received MLC-SLT for 52 weeks at doses of 180 mg bid (group A) or 120 mg bid (group B), or placebo (group C) for the first 26 weeks, switching at week 27 to receive MLC-SLT 180 mg bid (group C1) or 120 mg bid (group C2). No standard treatments or first-line medications for VaD were administered. There were two coprimary endpoints with patients assessed on VaDAS-Cog and ADCS-CGIC scales at baseline and at weeks 13, 26, 39, and 52. Secondary outcomes assessing global cognition, living ability, dementia severity, executive function, mental status, and behaviour, were evaluated on MMSE, ADCS-ADLs, and Clinical Dementia Rating (CDR) scale scores, with performance measured on the clock drawing task (CLOX) and the Chinese version of the executive interview (C-EXIT25), and the Neuropsychiatric Inventory (NPI). The secondary endpoint assessment was performed at baseline and at weeks 26 and 52, and brain MRI at baseline and week 52.

**FIGURE 4:**
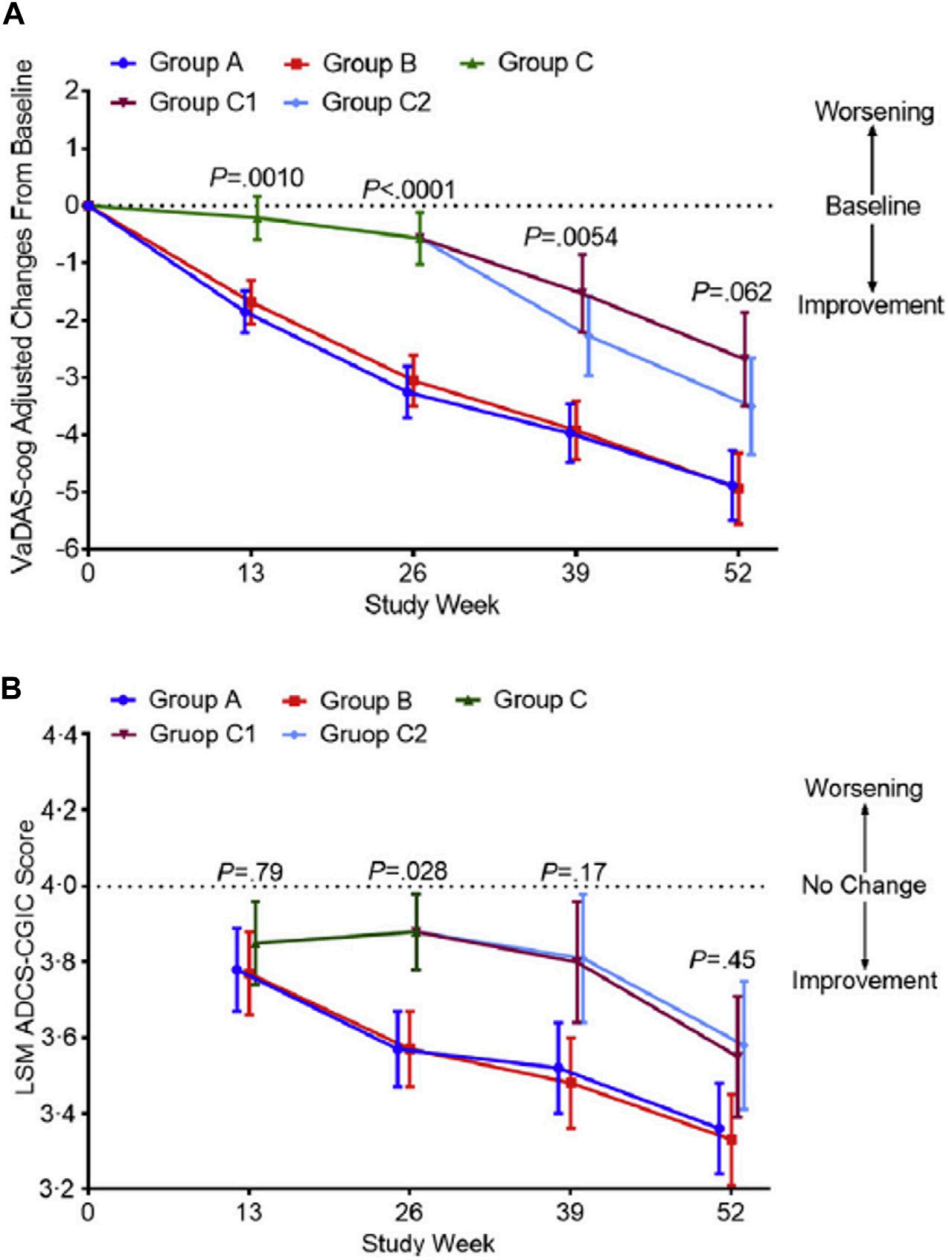
Changes in the VaDAS-cog and ADCS-CGIC scores from baseline to weeks 26 and 52 among the different groups. **(A)** The change in the VaDAS-cog score from baseline among groups was significantly different (*p* < 0.0001) at week 26. No significant difference was seen at week 52 (*p* < 0.062), confirming similar efficacy between the active and control groups after using SLT in the second 26 weeks of the study. **(B)** The change in the ADCS-CGIC score from baseline among groups was significantly different (*p* = 0.028) at week 26. Efficacy appears in groups C1 and C2 following use of SLT at week 52. Error bars are 95% confidence intervals. P represents the significance of the difference among groups. Abbreviations: VaDAS-cog, Vascular Dementia Assessment Scale–cognitive subscale; ADCS-CGIC, Alzheimer’s Disease Cooperative Study–Clinical Global Impression of Change; LSM, least squares mean; SLT, SaiLuoTong Changes in the VaDAS-cog and ADCS-CGIC scores from baseline to weeks 26 and 52 among the different groups from https://doi.org/10.1016/j.trci.2018.02.004 2352-8737/^©^ 2018 The Authors: Jianping Jia, Cuibai Wei, Shuoqi Chen, Fangyu Li, Yi Tang, Wei Qin, Lu Shi, Min Gong, Hui Xu, Fang Li, Jia He, Haiqing Song, Shanshan Yang, Aihong Zhou, Fen Wang, Xiumei Zuo, Changbiao Chu, Junhua Liang, Longfei Jia, Serge Gauthier. Published by Elsevier Inc. on behalf of the Alzheimer’s Association. This is an open access article under the CC BY-NC-ND license (http://creativecommons.org/licenses/by-nc-nd/4.0/).

Of the 340 included patients, 325 (96%) received at least one dose of study treatment with a safety assessment, and had at least one efficacy assessment after baseline. These patients were included in the safety population and modified ITT (mITT) population for efficacy. The assessment of cognitive and executive functions with VADAS-Cog scale showed a statistically significant improvement of cognitive and executive functions in both MLC-SLT groups A and B compared to placebo as early as 13 weeks, and at 26 weeks and through the end of the 1-year treatment follow-up (Figure 4A). At week 26, changes in VaDAS-cog scores (SD) from baseline were 23.25 (0.45) for group A (MLC-SLT 180 mg bid), 23.05 (0.45) for group B (MLC-SLT 120 mg bid) (both *p* < 0.0001), and 20.57 (0.45) for placebo (*p* = 0.15). The differences (95% CI) between both groups MLC-SLT compared to placebo were 2.67 (1.54–3.81) and 2.48 (1.34–3.62), respectively (*p* < 0.0001). There was no significant difference between MLC-SLT groups A and B.

The same was true for the scores observed on the other coprimary endpoint on ADCS-CGIC, with MLC-SLT improving the global condition of patients assessed by the clinician with input of their caregiver (Figure 4B). The divergence of outcomes between early-starter and delayed-starter groups was reversed as early as month 9 after switching to MLC-SLT in delayed-starter groups C1 and C2, with a sustained improvement up to month 12. In MLC-SLT group B, the proportion of patients with deteriorating function on ADCS-CGIC scale at month 6 was reduced by 47% compared to placebo. Concerning the secondary endpoints, most of them were significantly improved in MLC-SLT groups A and B vs. placebo group C at month 6. On the other hand, after the switch from placebo in group C to MLC-SLT in groups C1 and C2, the difference between delayed-starter (C1 and C2) and early-starter (A and B) groups was no longer significant. Regarding the safety profile, there was no significant difference in the rate of SAEs and AEs between the MLC-SLT and placebo groups, or between the early-starter and delayed-starter groups.

An exploratory analysis compared the difference in change of VADAS-Cog subscale scores between early- and delayed-starters over a 52-week period to assess a possible disease-modifying effect of MLC-SLT (Chen and Rahman, 2023). In the 325 patients from the mITT population, the VADAS-Cog change from baseline was compared between early-starters and delayed-starters by using linear mixed-effects models for repeated measures, with fixed effect of intervention (early-vs. delayed-starters), time, time and intervention interaction, baseline VADAS-cog and randomization stratification factors. An unstructured variance-covariance structure was used. Results showed that compared to delayed-starters, early-starters improved significantly adjusted mean change of VADAS-Cog score at all timepoints from week-13 (*p* < 0.001) to week 52 (*p* = 0.008) vs. baseline. Given that the delayed starters had not caught up with the early starters on the mean change in VADAS-Cog from baseline by week 52, this suggests that MLC-SLT may also have disease-modifying effects in addition to symptomatic effects.

#### 5.3.4 Efficacy and safety of sailuotong (MLC-SLT) in MCI

As pointed out by the authors of this trial (Steiner-Lim et al., 2023), MCI is a heterogeneous syndrome with cognitive decline accompanied by relative maintenance of ADLs. It is a prognostic factor for dementia and AD, increasing the risk 5 times. To date, there are no medicines licensed to treat MCI. Very recently, the results of a randomized, double-blind, placebo-controlled, parallel-group 12-week pilot phase II trial of Sailuotong (SLT) for cognitive function in older adults with MCI were published online in Alzheimer’s and Dementia: Translational Research & Clinical Interventions journal (Steiner-Lim et al., 2023). The 78 included participants aged 60 years and older with a MCI diagnosis, were randomly assigned to receive 180 mg/day capsule of MLC-SLT (n = 39) or a placebo (*n* = 39), of which 33 and 32 were analysed, respectively.

After 12 weeks, significant improvements were observed in MLC-SLT group compared to placebo group in primary outcome scores for Logical Memory delayed recall and the trail making test (TMT) condition 4 of Delis–Kaplan executive function (D-KEF). These results indicate a delayed recovery of episodic memory and improvement in executive function with respect to switching between cognitive concepts, higher-level divided attention, and multitasking in MLC-SLT group. Moreover, the maintenance of the spontaneous delayed recovery of unstructured verbal information assessed with Rey Auditory Verbal Learning Test (RAVLT) was achieved with MLC-SLT after 12 weeks, as shown by lower scores in the placebo group than in MLC-SLT one. Concerning activities of daily living assessed with functional activities questionnaire (FAQ), amazingly scores improved in placebo group but this change was not considered as clinically relevant.

Overall, these results showed that even after a relatively short treatment period of 12-week, MLC-SLT supports important aspects of memory and thinking in people with MCI and is well-tolerated.

#### 5.3.5 Summary of clinical development

The clinical development of MLC-SLT formulation has been conducted i) in healthy volunteers to first affirm its safety and then validating clinical translation of its preclinical properties, ii) in patients with VaD to confirm its efficacy and safety, and iii) in subjects with MCI.

Two studies in healthy volunteers have confirmed the safety and tolerability of MLC-SLT. One of these studies has also analysed its effect on neurocognitive function and cardiovascular (CV) parameters, showing favourable results on cognition and memory performance, and no CV safety issues.

Two clinical trials conducted in patients with VaD have demonstrated improvement of cognitive symptoms and activities of daily living, the main one with a delayed start design allowing to suggest a potential disease-modifying effect of MLC-SLT.

Furthermore, in its first placebo-controlled study in subjects with MCI, MLC-SLT demonstrated a statistically significant improvement in Logical Memory delayed recall scores vs. placebo and improved performance in executive function tasks. These findings are encouraging since such early intervention at the MCI stage may be critical in order to delay or prevent a diagnosis of dementia.

## 6 Discussion

At the end of this review of the pathophysiological and clinical continuum represented by the diagnosis of VCID and the arrival of MLC-SLT as a new therapeutic solution, we would like to draw some lessons from it.

With its incidence and prevalence probably underestimated due in particular to a complex diagnosis often confused and overlapped with that of AD, and although its ultimate form of VaD is already considered as the second cause of dementia, VCID is a public health issue that would deserve greater attention than it does today. Very few new drugs developed for dementia have randomized clinical trials targeting patients with VaD and even fewer for patients with VCI. Large pharmaceutical companies prefer to invest in the prevention of AD than in the treatment of VCID, although the latter also exerts huge morbidity costs, and lacks efficacious therapies (Linh et al., 2022). Meanwhile, given to the heterogeneous mechanisms of VCID (Yang et al., 2018), the increased difficulties have limited the development of its treatment.

More than two-thirds of people with dementia live in low- and middle-income countries (LMICs) (Kenne Malaha et al., 2023). For VCID, VCI on top of a stroke increases the economic burden and the burden of care, with the healthcare cost of VaD amounting to 23% higher than that of AD (Wimo and Winblad, 2003). In a community-dwelling population study about healthcare utilisation, it has been found that the highest annual cost of healthcare of US$14,387 was in VaD compared to US$7,839 for AD (*p* < 0.0001), the highest cost for hospital admissions being in VaD which had a 3-fold increase in number of hospital days compared to cerebrovascular disease without dementia (Hill et al., 2005).

One of the challenges in the management of VaD patients is to screen and diagnose them as early as possible. Often confused with Alzheimer’s disease or simply neglected in the shadow of aging, the diagnosis will be missed or overlooked, or even unknown. This represents a loss of opportunity for the patient and the initiation of treatment. Consensus groups attempt to bring together dispersed research initiatives into coordinated international programs, covering risk profile factors and various types of biomarkers such as cerebrospinal fluid proteins, structural and functional brain imaging, and genetic markers (Jaul and Meiron, 2017). So, the development of biomarkers for different forms of VCID remains a key issue (Custodero et al., 2022).

In recent years, the dementia world has been engulfed in the communication about the race for monoclonal antibodies (mABs) as disease modifiers in the early stages of AD and even MCI. All of this is underpinned by the significant developmental activity of disease-modifying therapies in AD, as shown in the 2023 annual review published by Cummings et al. (2023). The result is little room for VCID in research programs of large pharmaceutical companies, even in its most severe form of VaD (Wang et al., 2023). It is in this environment that the market release of MLC-SLT in Singapore took place in October 2022. The results of the preclinical development of MLC-SLT have confirmed its multi-target mechanisms of action simultaneously improving cerebral vascularisation and cognitive disorders. Studies in healthy volunteers have confirmed its safety and tolerability, as well as its favourable effects on neurocognitive function and CV parameters. cognition, and memory performance. In two small clinical trials conducted in patients with VaD (Jia et al., 2018; Liu et al., 2022), MLC-SLT improved cognitive symptoms and activities of daily living, with good safety results. It has potential for disease modification that should be confirmed. Recently, the positive results of a clinical study with MLC-SLT in patients with MCI (Steiner-Lim et al., 2023), expand its place in the management of VCID. MLC-SLT (SaiLuoTong) is a useful and promising addition by taking part in the success of the struggle against VAD, which remains limited to date.

## Figures and Tables

**Table:** 

**Aβ peptide**	amyloid β peptide
**AChEI**	acetylcholinesterase inhibitor
**AD**	Alzheimer’s disease
**ADAS-Cog**	Alzheimer’s Disease Assessment Scale-Cognitive subscale
**ADCS-ADL**	Alzheimer’s Disease Cooperative Study–Activities of Daily Living
**ADL**	activities of daily living
**bid**	two times a day
**Cs**	Crocus sativus
**CSVD**	cerebral small vessel disease
**CT**	computed tomography
**CVRFs**	cardiovascular risk factors
**DSM-V**	Diagnostic Manual of Mental Disorders, Fifth Edition
**FBV**	foetal bovine serum
**Gb**	Ginkgo biloba
**GFAP**	glial fibrillary acidic protein
**H2O2**	Hydrogen peroxide
**ITT**	intention to treat
**L-NAME**	l-N-nitro arginine methyl ester
**LCN2**	lipocalin 2
**MCI**	mild cognitive impairment
**mITT**	modified ITT
**MLC-SLT**	SaiLuoTong
**MMSE**	mini mental-state examination
**MRI**	magnetic resonance imaging
**Pg**	Panax ginseng
**p-JAK2**	phosphorylated Janus kinase 2
**p-STAT3**	phosphorylated signal transducer and activator of transcription 3
**ROS**	reactive oxygen species
**SF-36**	Short Form Health Survey-36
**SOD**	superoxide dismutase
**SPECT**	Single Photon Emission Computed Tomography
**TCM**	traditional Chinese medicine
**t.i.d**	three times a day
**TZ**	Tianzhi
**VaD**	vascular dementia
**VADAS-Cog**	Vascular Dementia Assessment Scale-Cognitive subscale
**VCI**	vascular cognitive impairment
**VCID**	vascular cognitive impairment and dementia
**WMLs**	white matter lesions
